# The influence of conversational agents’ role and communication style on user experience

**DOI:** 10.3389/fpsyg.2023.1266186

**Published:** 2023-12-01

**Authors:** Remi Poivet, Mélanie Lopez Malet, Catherine Pelachaud, Malika Auvray

**Affiliations:** ^1^Ubisoft Paris Studio, Paris, France; ^2^Institut des Systèmes Intelligents et de Robotiques (ISIR), Sorbonne Université, Paris, France

**Keywords:** conversational agents, verbal interactions, human behaviors, human-machine interaction, believability

## Abstract

Conversational Agents (CAs) are characterized by their roles within a narrative and the communication style they adopt during conversations. Within computer games, users’ evaluation of the narrative is influenced by their estimation of CAs’ intelligence and believability. However, the impact of CAs’ roles and communication styles on users’ experience remains unclear. This research investigates such influence of CAs’ roles and communication styles through a crime-solving textual game. Four different CAs were developed and each of them was assigned to a role of either witness or suspect and to a communication style than can be either aggressive or cooperative. Communication styles were simulated through a Wizard of Oz method. Users’ task was to interact, through real-time written exchanges, with the four CAs and then to identify the culprit, assess the certainty of their judgments, and rank the CAs based on their conversational preferences. In addition, users’ experience was evaluated using perceptual measures (perceived intelligence and believability scales) and behavioral measures (including analysis of users’ input length, input delay, and conversation length). The results revealed that users’ evaluation of CAs’ intelligence and believability was primarily influenced by CAs’ roles. On the other hand, users’ conversational behaviors were mainly influenced by CAs’ communication styles. CAs’ communication styles also significantly determined users’ choice of the culprit and conversational preferences.

## 1 Introduction

During interactive experiences, conversational agents (CAs) convey the narrative through their interactions with users. Designers can manipulate several parameters to influence users’ experience of the narrative. For instance, users can be told the explicit roles of CAs in the narrative, allowing them to adjust more optimally their conversational strategies. Moreover, CAs’ communication style is crucial in the interaction as it determines the form of the content transmitted to users. Additionally, research on human-agent interactions demonstrated the importance of considering users’ perception of intelligence and believability attribution to create more engaging agents (Loyall, 1997; Bartneck et al., 2009). This study aims at providing insights into the influence of CAs’ role and communication style on users’ experience. A textual computer game was created in which users endorse the role of a detective investigating a case. They were requested to engage in conversations with four CAs and to name a culprit among them. CAs could have different roles (witness or suspect) and communication styles (aggressive vs. cooperative). Users’ evaluation of CAs’ was measured through items of perceived intelligence and believability scales and users’ behaviors were analyzed through the measure of their input length (number of characters in their message), delay (number of seconds to send their message), and conversations length (number of turns in their conversations).

In the remainder of the manuscript, we first highlight the importance and impacts of CAs’ roles and communication style in narrative environments. Then, we describe our experimental approach, outlining the narrative setting scenario and the manipulation of CAs’ parameters. Next, we present and analyze the findings, before concluding the paper with our final remarks.

## 2 Background

Conversational Agents, also known as chatbots, are a type of artificial agents that aims to simulate human conversation through natural language processing and generation (Klüwer, 2011). In computer games, the number of textual narrative games is growing and so is the importance of CAs in their design. These games offer an interactive experience that relies on textual communication between the user and different CAs. Effective communication is crucial for users’ immersion and engagement in the narrative (Isbister and Nass, 2000), as it directly impacts their enjoyment of the game (Schaffer and Fang, 2019). Therefore, designing CAs requires careful consideration of users’ perception and expectations.

Conversational Agents involved in narrative experiences are characterized by their role and their communication style toward the user. These factors aim to influence users’ expectations, their evaluation of the agent, and their conversations (Mou and Peng, 2009; Nag and Yalçın, 2020). Users’ expectations are influenced by their knowledge of CAs (Komatsu and Yamada, 2011) and by implicit stereotypes, which are associated with positive evaluations or negative ones (Brahnam and De Angeli, 2012).

Regarding roles, in narrative experiences, CAs can assume, for example, either friendly roles or opponent ones toward the user. These roles influence the agents’ function in the narrative. For instance, an opponent’s role would convey the challenge by their antagonistic attitude in the narrative experience. Information about the role can be explicitly communicated (e.g., the agent is introduced by the narrator as an opponent or as an ally) or inferred by users during their interaction (e.g., through the agent’s communication style). Explicit roles aim to shape users’ interactions by triggering pre-existing positive or negative stereotypes before the actual conversation occurs. For instance, the explicit role of an opponent affects users’ expectations, which involves an anticipation of their interaction influenced by the stereotypes associated with hostility. These expectations hence shape users’ decision to interact with the CAs (e.g., adopting an appropriate communication strategy or even, avoiding the interaction).

Conversational Agents are also characterized by their communication style. This includes content generation, conversational strategy, and linguistic cues to convey CAs’ intentions and personality traits (van Pinxteren et al., 2023). In narrative experiences, before an interaction with CAs, users form expectations of their communication style based on the stereotypes associated with CAs’ explicit roles. For instance, users are likely to expect an opponent to adopt an aggressive communication style, since this trait is associated with hostility (Infante, 1995). Therefore, designing communication style is crucial for users’ interactions, which involves the choice of CAs’ conversational strategy and the choice of linguistic cues (Mairesse and Walker, 2009; Resendez, 2020). For instance, designers of CAs can use linguistic cues associated with the personality traits of the Big Five model to convey distinct personalities, such as extraversion, conscientiousness, agreeableness, openness to experience, and neuroticism (Mairesse and Walker, 2007). For example, a CA using a formal lexicon conveys more conscientiousness to users but less extraversion than a CA with an informal one (Heylighen and Dewaele, 2002). Personality traits can also affect users’ behaviors and engagement during their conversations. Ruane et al.’s (2021) study examined the impact of chatbots’ perceived personality on users’ engagement and preference. The authors hypothesized that users would engage in longer conversations with their preferred CA. They created two versions of chatbots using linguistic cues associated with the Big Five personality model: one with high extraversion and agreeableness and the other with the opposite traits. The study measured participants’ input length (number of words in their messages) and conversation length (number of minutes and turns), and found that participants tended to mimic the linguistic cues of the chatbot they were interacting with. In this context, the formal lexicon associated with the low extraversion of the second chatbot led to longer conversations. However, participants preferred their conversations with the first chatbot as it was perceived as more agreeable. In conclusion, the selection of appropriate linguistic cues and adjustment to the content of communication is crucial to create CAs with different personalities and engaging qualities (Følstad and Skjuve, 2019).

Creating opponent and friendly CAs involves different requirements. The communication style of opponent CAs has to be perceived as aggressive to accurately convey their intention. Their conversational strategy relies on verbal aggressiveness, which reflects an intention to attack the interlocutor. The desired outcome involves emotionally affecting the interlocutor, for example by inducing humiliation and negative feelings (Infante and Wigley, 1986). For instance, to operationalize the strategy, the negative content polarization, the lexicon formality, and the use of swear words are associated with aggressive ascriptions (Pennebaker and King, 1999; Mehl et al., 2006). Overall, an aggressive communication style conveys a disagreeable personality trait (Mairesse and Walker, 2009) which reflects the distinction between expected personalities and outcomes with opponent or with friendly roles in a narrative experience. Users expect opponents in the narrative to induce negative consequences based on their stereotypes, and thus agents are expected to be disagreeable and their communication style to be aggressive.

On the other hand, the communication style of friendly CAs involves linguistic cues associated with positive personality traits such as agreeableness, openness to experience, or extraversion (Völkel et al., 2020) and a friendly conversational strategy (Simpson et al., 2020). Friendly CAs would be more inclined to positive content polarization and less inclined to negative topics (Mehl et al., 2006). For instance, a friendly CA in a narrative would be expected to diffuse tensions during their interactions with users using consilience markers (e.g., generate apologies during misunderstandings with users, see de Sá Siqueira et al., 2023). Moreover, friendly CAs are associated with higher extraversion and they are more inclined to larger verbosity and informal lexicon (Mairesse and Walker, 2009). As a result, combining the relevant linguistic cues and conversational strategy for a friendly communication style aims at having friendly CAs more engaging and cooperative during their interactions with users.

## 3 Scales of perceived intelligence and believability

During interactive experiences, CAs’ perceived intelligence and believability attributions are associated with users’ enjoyment and motivation to interact (Loyall, 1997; Moussawi et al., 2021). Therefore, understanding how the role and communication style of CAs affect these attributions could greatly enhance their design, and thus users’ experience. Two scales are particularly relevant to do so: Perceived intelligence and Believability.

Perceived intelligence can be used to probe users’ evaluation of CAs’ intelligence (Bartneck et al., 2009). Such evaluation relies on two dimensions: understandability and performance (Koda and Maes, 1996), both based on users’ understanding of the agents’ purposes and efficiency in reaching their goals. For instance, CAs’ perceived intelligence would rely on their capacity to accurately simulate natural human communication with users. However, in narrative experiences, CAs’ role can affect users’ attitudes, and thus influence their expectations of the agents’ communication style and purpose. In that sense, the perceived intelligence of a friendly CA or an aggressive one is different, as their purposes in the narrative are not the same. Namely, friendly agents are conceived to help users, while aggressive ones are meant to increase the challenge of the game. To improve artificial agents’ design by considering users’ perception, Warner and Sugarman (1986) proposed an intelligence evaluation scale that relies on five semantic items: Incompetent/Competent, Ignorant/Knowledgeable, Irresponsible/Responsible, Unintelligent/Intelligent, Foolish/Sensible. This scale assesses users’ judgment of the two dimensions of understandability and performance.

Besides intelligence perception, when users believe in their interaction with CAs their level of engagement increases (Nag and Yalçın, 2020). As these agents aim to simulate human communication, it is necessary to understand how their design conveys believability from the user’s point of view. As users evaluate agents from their expectations of how they should behave, believable agents ought to have a close correspondence between users’ expectations and their interaction (Loyall, 1997). Therefore, agents’ roles can influence users’ evaluation as they expect these agents to have different purposes (e.g., interactions will then differ when occurring with friendly or aggressive agents). Gomes et al. (2013) developed a scale comprising multiple dimensions which play crucial roles in determining how interactive agents are perceived as believable in narrative experiences. These dimensions listed below allow one to quantify agents’ believability through users’ ratings (Where <X> is replaced by the evaluated agent).

• Awareness: <X>perceives the world around him/her.
• Behavior understandability: It is easy to understand what <X> is thinking about.
• Personality: <X> has a personality.
• Visual impact: <X>’s behavior draws my attention.
• Predictability: <X>’s behavior is predictable.
• Behavior coherence: <X>’s behavior is coherent.
• Change with experience: <X>’s behavior changes according to experience.
• Social: <X> interacts socially with other characters.

Understanding the effects of the agents’ design parameters on believability and on perceived intelligence could help designers conceive more engaging narrative agents.

To summarize, CAs’ design is crucial for positive users’ experiences, as these agents convey the core of the narrative. CAs’ role can influence users’ expectations and attitudes toward them, while the communication style impacts their behaviors and their perception of the agents’ personality traits. Previous research has shown that studying users’ perceived intelligence and believability of artificial agents can provide crucial information to improve designers’ choices. However, how these design factors affect users’ experience remains unclear. This research aims at investigating how CAs’ roles and communication styles affect users’ ascription of intelligence and believability. To do so, an experiment was conducted in which participants interact with different CAs set in a detective game scenario.

## 4 General methods and procedure

**FIGURE 1:**
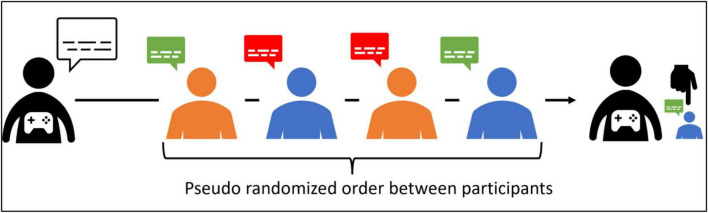
Example of a participant’s session: aggressive and co-operative communication styles are represented in red and in green, respectively. Then, the roles of witness and suspect are represented in orange and blue, respectively. Participants are asked to identify the culprit among the four CAs after a conversation with each of them.

A French computer textual game was developed for this experiment in which participants endorse the role of a detective. Their task consisted in interacting through conversations with four CAs and then to name a culprit among them. The CAs were defined by their identity, personality traits, and knowledge about the case. Before each discussion, the explicit role of the CA was given to the detective. The participants interacted with the four CAs. To avoid biases, for each participant, each CA (Anthony Frey, the co-manager; Enzo Lamy, the barman; Mathieu Fournier, the croupier and Christian Vigneron, the security agent) was assigned pseudo-randomly a role (suspect vs. witness) and a communication style (aggressive vs. cooperative) (see Figure 1). This ensured that each participant experienced all the experimental conditions. To generate the CAs’ answers, a Wizard of Oz (WoZ) selected outputs from a predefined list of sentences. To prevent disruptions in the conversation when participants ask unanticipated questions and to generate coherent answers, the WoZ was given access to characters’ personal information and knowledge about the scenario. The duration of each discussion was set to 10 min. The investigation of users’ experience involved participants’ rating on items of perceived intelligence (Bartneck et al., 2009) and believability (Gomes et al., 2013) scales after each discussion. The lexicon of the believability scales was modified to suit the interaction context, replacing the term “behavior” in the various items with “discourse.” Additionally, participants rated their perception of warmth (from “0–very cold” to “100–very warm”) and cooperation/aggressivity (from “0–cooperant” to “100–aggressive”). Participants’ behaviors were analyzed through the measure of their inputs’ length (number of characters in their sentences) and delay (number of seconds to send their input), and the number of turns during their conversation. After the four interactions, participants were required to indicate a culprit and to rank the conversations, with the first place indicating their most preferred conversation and the fourth place representing the least preferred one.

### 4.1 Participants

Thirty-two French employees of Ubisoft participated in the experiment, comprising 19 men and 13 women, with an average age of 29 years (SD = 7.3). The participants rated their frequency of playing video games and role-playing games using scales ranging from “0–I never play” to “100–I play every day.” There was a gender difference in role-playing game habits, with women reporting a mean of 31.4 (SD = 29.8) and men reporting a mean of 54 (SD = 32.9), while no difference was found in video game habits (mean = 73.9, SD = 25.1). All participants were contacted via email and they were provided with information regarding the general purpose of the research. The email emphasized the voluntary nature of participation and the option to withdraw at any time. The experiment lasted approximately 50 min and was conducted following the principles outlined in the Declaration of Helsinki.

## 5 Materials and methods

**FIGURE 2:**
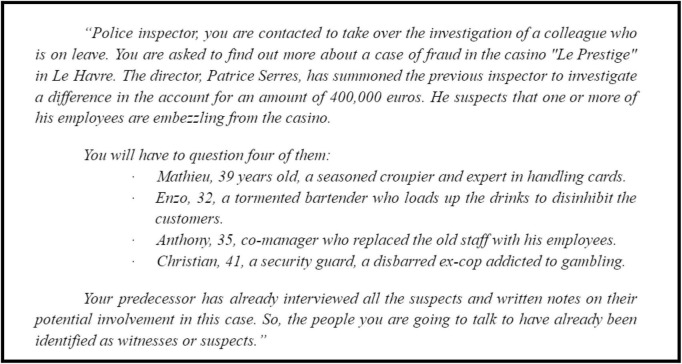
The scenario of the game communicated to participants.

The scenario of the game consists in a police investigation case in which participants endorse the role of a detective and have to discuss with four CAs to solve the crime case (see Figure 2). Before each interaction, participants were provided with contextual information regarding the police investigation and CAs’ identities. This information sets the narrative of the game and aims to guide participants’ inquiries during their discussions with the CAs.

The four CAs are defined by their identity, personality traits, and knowledge about the crime. Their identities involve CA’s personal information (i.e., agent’s name, age, and profession) and their backstories that aim to add in-depth details. For instance, one of the CAs, Christian, is described as a 41-year-old man, working as a security agent in the casino. Each CA’s identity was ascribed personality traits from the Big Five model and was communicated through linguistic markers. The purpose of describing each identity with different personality traits is to create diverse and engaging CAs by giving them distinct but consistent personalities. For instance, Christian is high in extraversion but low in conscientiousness as he will be more likely to use informal a lexicon, slang language, and in-group markers such as “my pal” when speaking with the detective (see Table 1 for the details of all the personality traits and the associated linguistic cues). Additionally, CAs’ gender was the same (here male, to avoid stereotypes on gender-aggressivity association). Knowledge about the crime refers to the content CAs can communicate to participants. For instance, Anthony, as the manager of the casino, can provide information about his employees’ enrollment. On the other hand, Enzo, as barman of the casino, is more likely to discuss details about clients that he might have collected through former conversations with them. Moreover, the relationships between the CAs were defined beforehand and controlled in their content, as social interactions between witnesses and suspects are important in a crime-solving situation (e.g., to name a culprit, the detective might rely on the relationship between the suspects). Each identity is described below.

**TABLE 1:** Linguistic markers associated with each identity’s content generation.

Agent’s identity	Linguistic markers
Mathieu Fournier (39 years old, Croupier)	Medium verbosity Contracted negation (e.g., “I can’t tell you…”) Informal lexicon (slight vulgarity, e.g., “it pisses me off when…”) Impolite form of address (sarcastic tendency, e.g., “Yeah, it’s the colonel mustard who did it.”)
Enzo Lamy (32 years old, Barman)	Strong verbosity Contracted negation (e.g., “I can’t tell you…”) Formal lexicon (no vulgarity unless under accusation in aggressive verbal behaviors) Polite form of address Emotional reaction (e.g., “oh no…,” “I’m so anxious about…”)
Anthony Frey (35 years old, Co-manager)	Strong verbosity Un-contracted negation (e.g., “I do not…,” “I cannot…”) Formal lexicon (no vulgarity and rich vocabulary, e.g., “This accusation is outrageous”) Polite form of address
Christian Vigneron (41 years old, Security guard)	Medium verbosity Contracted negation (e.g., “I can’t tell you…”) Informal lexicon (slang and swear words) Impolite form of address (in-group markers, e.g., “I get you, my pal.”)

- *Mathieu Fournier*, 39 years old, croupier. Mathieu is the oldest employee of the casino. He has been in the job for 15 years and is well-liked by the customers. He is described as very skilled by his coworkers. Mathieu is high in extraversion but low in agreeableness as he tends to use an informal lexicon and an impolite form of address. Thus, he has a quick wit and a dry sense of humor. He sees Anthony as a very ambitious person and since his arrival as co-manager, he now has Christian at his table who acts as a fake player in the games. He doesn’t like his presence as he feels watched but remains professional. He spends his free time at Enzo’s counter without necessarily talking to him. He has a positive opinion of Enzo’s competence in his profession.

- *Enzo Lamy*, 32 years old, barman. Enzo is a mixology enthusiast and is confronted with unpleasant behavior from drunk customers. In addition, he respects the alcohol dosages instructions given by the management which indicates a high conscientiousness. However, he is high in neuroticism, which involves anxious reactivity during his interactions with the detective. He has been hired by Anthony; they have a relationship of trust. He does not talk much to Mathieu although he finds him competent. He appreciates Christian’s unconventional personality.

- *Anthony Frey*, 35 years old, co-manager. Anthony is a highly educated man. As such, he uses a formal lexicon and adopts a polite form of address toward the detective. He was the one discovering the error in the accounts and notified the police. He is high in conscientiousness as he changed procedures and replaced staff before notifying the police. He recruited Christian, whom he finds useful to the casino despite their very different personalities. He recruited Enzo, whom he finds very competent in his work and contributes to a good customer experience. Mathieu is the only employee who has not been replaced by Anthony, hence he does not know him well but has nothing against him.

- *Christian Vigneron*, 41 years old, security agent. Christian is a former police officer who was disbarred for alcohol and gambling problems. Christian is high in extraversion but low in conscientiousness as he tends to use familiar lexicon and in-group markers when he interacts with the detective. He has been recruited by Anthony, who he likes although he considers him to be his opposite personality. He spends time at Mathieu’s table as a fake gambler to keep an eye on the customers, thanks to an envelope given to him by the management. He thinks Mathieu is a good croupier and has nothing against him. He spends the rest of his time sitting at Enzo’s bar. Mathieu appreciates that Enzo is a good listener.

Conversational Agents’ communication styles were manipulated to convey aggressive or cooperative intentions. To do so, hostility and agreeableness markers based on communication theory (Infante, 1995; Pennebaker and King, 1999; Mairesse and Walker, 2007; Mairesse and Walker, 2009) have been implemented in the communication content to affect participants’ evaluation of aggressivity and cooperation. On the one hand, CAs in their aggressive form had less verbosity, used personal attacks, and had negative content polarization. Moreover, they had aggressive sentences toward the participants rather than answering their inquiries (e.g., “Do you even know what you are talking about?”). On the other hand, CAs in their cooperative form had more verbosity and answered pedagogically to the detective’s questions. In addition, agents with a cooperative communication style requested confirmation for the relevance of their answers (e.g., “I hope my answers will help you solve this affair”) and used consilience markers (e.g., “sir” or “detective”).

To control the form of CAs’ communication style, a Wizard of Oz (WoZ) method was used. Precisely, the WoZ used a working sheet for each identity (see Table 2 for an example of Anthony’s identity). This working sheet consisted in a list of the detective’s potential questions and the content’s communication declension. In particular, the content communication could come in two styles (aggressive or cooperative) that the WoZ followed based on the experimental condition the discussion was set in (e.g., an aggressive or a cooperative CA). The potential questions asked by participants were listed based on the intention associated and involved specific situations such as “Initial contact,” “Backstory information,” and “Accusation.” If participants asked follow-up questions about a specific topic, the WoZ either rephrased their answer in the cooperative form condition or made the answer more aggressive (i.e., the Wizard of Oz answers the question and adds impatience markers such as “as I already said,” “Your questions are annoying”).

**TABLE 2:** Example from the Wizard of Oz’s working sheet: Anthony, the co-manager’s answers.

Detective’s potential question	Question’s intent	Cooperative answers	Aggressive answers
*“Hello.”*	Initial contact	*“Hello sir.”*	*“Hello sir.”*
*“How are you?”*	Initial contact	*“Personally, I am doing very well. Even though the disappearance of such a large sum of money is on my mind.”*	*“Let’s get to the point, why am I being questioned about the disappearance of 400,000 euros in my own casino?”*
*“How long have you been working here?”*	Backstory information	*“It has been 2 years since I was hired to modernize the casino ‘Le Prestige.’ The old casino was obsolete and this modernization with the addition of the hotel complex makes it possible to make this place a splendid jewel for tourism.”*	*“I’ve been the co-manager of the casino for 2 years. But if the goal is to have such basic information, go to HR, you will waste less time.”*
*“Have you stolen the money?”*	Suspicion	*“Of course not. I am the one who warned the police about the discrepancy in the accounts, I think that clears me. But if some elements are not clear to you, I am ready to answer all of your questions.”*	*“This accusation is outrageous! Do your job seriously before you come and waste my time.”*
*“Are you the culprit?”*	Suspicion	*“I am not. On the contrary, I’m here to help you find out who the real culprit is.”*	*“Of course not! I’m looking for it, just like you. So do your job!”*
*“I have some evidence against you!”*	Accusation	*“What evidence are you talking about? In any case, I am ready to tell you everything I know.”*	*“This attempt at destabilization is ridiculous. What evidence? Be specific!”*
*“You look nervous.”*	Follow-up	*“I’m not. As an exemplary leader, I work under constant pressure. What you call nervousness, I call responsiveness.”*	*“I’m just annoyed by the level of your questions. I have several meetings today so make it quick and better.”*
*“What do you think of Mathieu?”*	Social interaction	*“He seems to be impeccable. Clients like him and he has adapted well to the change in staff.”*	*“He seems experienced.”*
*“Thanks for your answers.”*	Gratitude	*“Thanks to you. I remain at your service.”*	*“I wouldn’t say that the pleasure is shared.”*

The CAs’ roles in the scenario are closely tied to their context (i.e., a witness or a suspect). For instance, in a crime-solving game, witnesses can be expected to act as cooperative agents who assist participants in solving the crime through their communication, while suspects can be expected to be more hostile and convey their motivation in the game (i.e., indicating whether they are guilty or not). Before each discussion, participants were given a brief description of the CA’s identity content (i.e., the same description of the experiment’s introduction) and their role in the scenario (e.g., “Witness: Enzo, 32 years old, a tormented bartender who loads up the drinks to disinhibit the customers”).

## 6 Results

The analysis of participants’ interactions with the four CAs includes CAs’ evaluation through rating scales and behavioral measures of the conversations. Rating scales consisted of each of the items on perceived intelligence and believability scales, to which were added specific items to collect judgment of CA’s warmth and aggressiveness (see section “4. General methods and procedure”). The behavioral measures included the participants’ input length, the delay, and the number of turns during conversations. Finally, the culprit’s designation and preference’s ranking were analyzed.

A three-way ANOVA was conducted on participants’ ratings of their conversations with CAs. Due to the multiple items involved in the scales, only the statistically significant ones are reported here. The factor “role” had a significant impact on the item “Visual impact” of the believability scale (i.e., “ <X>’s discourse draws my attention”). When CAs were introduced as witnesses, participants were significantly more attentive during the conversation compared to suspects [*F*_(1, 124)_ = 5.147, *p* = 0.025]. Furthermore, when examining the different levels of the “communication style” factor, a simple effect analysis of the “role” factor revealed that participants rated their attention significantly lower when suspects exhibited a cooperative communication style compared to an aggressive one (*p* = 0.016). The “communication style” factor had a significant impact on participants’ rating of warmth [*F*_(1, 124)_ = 34.086, *p* < 0.001] and aggressivity [*F*_(1, 124)_ = 258.903, *p* < 0.001]. The interaction between the “role” and “order” factors had a significant effect on participants’ ratings. Precisely, there were significant differences in the evaluations of the first and last encountered CAs. The analysis indicated that participants rated their attention higher when CAs were introduced as witnesses [*F*_(1, 28)_ = 4.773, *p* = 0.037] and attributed to them more personality than to suspects [*F*_(1, 28)_ = 10.817, *p* = 0.003]. Suspects were evaluated as more competent [*F*_(1, 28)_ = 9.789, *p* = 0.004], knowledgeable [*F*_(1, 28)_ = 18.640, *p* < 0.001], intelligent [*F*_(1, 28)_ = 14.497, *p* < 0.001], sensible [*F*_(1, 28)_ = 7.846, *p* = 0.009], and responsible [*F*_(1, 28)_ = 4.443, *p* = 0.045] than witnesses. The interaction between order and communication style had no significant impact on participants’ ratings.

Regarding participants’ behaviors, each of the three measures described above was analyzed separately using three-way ANOVAs following the same approach of the participants’ ratings of the items’ scales. Regarding the input length, the results showed a significant effect of the factor “communication style” [*F*_(1, 1067)_ = 5.017, *p* = 0.025]. Participants made longer inputs (in terms of sentence length) when they were interacting with aggressive CAs. There was a significant interaction between the factors “role” and “order” [*F*_(3, 1067)_ = 3.829, *p* = 0.010]. Participants wrote sentences with more characters when interacting with suspects during the first and second conversations, while it is the opposite for the last conversations. There was also a significant interaction between the factors “role,” “communication style,” and “order” [*F*_(3, 1067)_ = 3.287, *p* = 0.020]. Precisely, the order of the conversation had a significant impact on participants’ input length for aggressive suspects (*p* = 0.029). Regarding the delay of the inputs, the analysis excluded participants’ first message, as it initiated their conversation. The results of the three-factor ANOVA indicated a significant interaction between the factors “role” and “order” [*F*_(3, 1067)_ = 8.071, *p* < 0.001]. Participants took longer to write their inputs when they faced suspects during the first and second conversations, while it is the opposite during the last conversations. There was a significant difference between the factors “role,” “communication style,” and “order” [*F*_(3, 1067)_ = 6.216, *p* < 0.001]. Simple main effects analyses showed that the order of the conversation had a significant impact on participants’ delay for aggressive suspects (*p* = 0.002), cooperative suspects (*p* < 0.001), and aggressive witnesses (*p* = 0.009), but no significant effect was observed for cooperative witnesses (*p* = 0.370). Finally, the analysis of the number of turns during the conversations revealed a significant impact of communication style [*F*_(1, 1064)_ = 64.494, *p* < 0.001] on conversations. Conversations with aggressive CAs were significantly longer than those with cooperative ones. There was an effect of order [*F*_(3, 1064)_ = 6.574, *p* < 0.001], with participants having longer conversations by the last encountered CAs. There was a significant interaction between order and role [*F*_(3, 1064)_ = 10.546, *p* < 0.001] as conversations with suspects were longer when they were encountered last. There was a significant interaction between order and communication style [*F*_(3, 1064)_ = 4.762, *p* = 0.003] highlighting a significant difference between aggressive and cooperative CAs through the conversations. Lastly, there was a significant interaction between order, role, and style [*F*_(3, 1064)_ = 10.598, *p* < 0.001]. Simple main effects revealed that the order of interaction influenced the conversations’ length for aggressive suspects (*p* < 0.001), cooperative suspects (*p* < 0.001), and aggressive witnesses (*p* = 0.011). Notably, conversations with cooperative witnesses did not appear to be affected by the order of the conversation.

Regarding participants’ indication of the culprit and their ranking of the conversations. A contingency table was used to analyze the distribution of participants’ indications of the culprit across the four conditions. A chi-squared test was conducted, and the results did not show any significant difference between witnesses and suspects (χ^2^ = 0.439, *p* = 0.508) but they revealed a significant effect of the “communication style” (χ^2^ = 4.176, *p* = 0.029). In addition, a linear regression analysis was conducted on participants’ certainty scores to identify predictors of their choice of the culprit. The regression only identified the aggressivity score as a significant predictor of participants’ certainty (*r* = 0.553, *p* = 0.009). A log-linear regression was conducted to analyze the relationship between “role,” “communication style,” and participants’ ranking of conversations. The analysis only indicated a significant association between “communication style” and participants’ preference [*r* = 0.170 (SE = 0.057), *z* = 3.004, *p* = 0.003]. A MANOVA was conducted on participants’ ratings and behavioral measures, with the ranking of the conversation as a factor. The analysis indicated a significant difference in the scores of the items “predictability” [*F*_(1, 124)_ = 2.813, *p* = 0.042] and “aggressiveness” [*F*_(1, 124)_ = 5.143, *p* = 0.002]. The most and least preferred conversations were rated as highly predictable, with the preferred one rated as less aggressive than the least preferred one. The length of the conversation was the only significant effect on participants’ ranking [*F*_(3, 1079)_ = 3.621, *p* = 0.013]. The preferred conversation was significantly longer compared to the other ones.

## 7 Discussion

Our experiments, by combining participants’ ratings of perceived intelligence and believability with their behaviors during conversations, allow obtaining a comprehensive understanding of how CAs’ design influence user’s experience in a detective computer game. In particular, our results revealed five main results, (1) The design of CAs influences participants’ ratings of perceived intelligence and believability. (2) CAs’ communication styles play a crucial role in shaping participants’ perception of aggressiveness and warmth. (3) These communication styles also influence participants’ behaviors, such as the size of their inputs and the frequency of turns taken during the conversation. (4) Participants’ preferences for the conversations are closely related to CAs’ communication styles. (5) In light of these findings, it becomes obvious that aligning the roles of CAs with their communication styles has the potential to significantly improve users’ experience. These findings are discussed in the following subsections.

Finding 1 revealed the influence of CAs’ roles on participants’ ratings of perceived intelligence and believability, regardless of their communication style. The explicit roles (suspect vs. witness) aim to activate stereotypes in participants’ minds, and thus generate expectations. In a police investigation situation, suspects and witnesses are known to engage in distinct types of interactions with investigators. Interactions with suspects typically involve highly challenging and argumentative conversations, as suspects are expected to defend their alibis. On the contrary, witnesses readily provide crucial information to facilitate the progression of the investigation. In our experiment, the roles were explicitly communicated to participants before they interact with CAs, allowing them to anticipate the conversations they might have and consequently their conversational strategies. These strategies include predicting the topic of the detective’s inquiries and adjusting their approach accordingly. In our experiment, the strategies adopted by the participants may have influenced their evaluation of the encountered CAs, particularly during the first interaction. Using the item “Visual impact” of the believability scale to rate the attention drawn by CAs’ discourse (“<X>’s discourse draws my attention”), participants’ attention was higher when they were interacting with witnesses. Participants were found to be more attentive to the discourse of witnesses. Subsequently, participants rated the perceived intelligence of suspects significantly higher than that of witnesses. When conversing with suspects, participants were more inclined toward suspicion and accusatory inquiries, while conversations with witnesses tended to be more informative in nature. The different strategies adopted by participants reflect their underlying motivation during the interaction, as suspects are implicitly more likely to be identified as the culprit. Participants may have perceived the suspects as being more intelligent because they responded to accusations and suspicions, whereas the strategy used toward witnesses involved informative inquiries, which typically resulted in less argumentative responses. These different strategies appeared obvious in participants’ behaviors as their input length and delay were affected by the roles and order of CAs. In addition to the effect of role, participants changed of behaviors across conversations. In particular, they wrote longer inputs and took more time to formulate their inquiries when they encountered a suspect before any witness. Conversely, participants adopted the opposite approach when interacting with witnesses during the last conversations. This shift in strategy suggests that participants initially prioritized interactions with suspects, anticipating more crucial information to help them discover the culprit.

Finding 2 confirms the significant effect of the linguistic cues and the strategies used to convey CAs’ communication style and its impact on participants’ evaluation (Mairesse and Walker, 2009; Resendez, 2020). CAs with an aggressive communication style were perceived as more aggressive and colder in comparison to cooperative ones (Infante and Wigley, 1986). Although there was no significant effect of communication style on participants’ perception of intelligence and believability, the communication style did impact the item “Personality” of the believability scale regarding the suspects.

Finding 3 underscores the effect of CA’s communication style on participants’ behaviors, reflected by their inputs and the number of turns during the conversations. These findings diverge from those obtained by Ruane et al. (2021). In their study, participants tended to mimic the communication style of chatbot as they would write longer sentences and have deeper conversations with chatbots using extraversion communication style (i.e., writing longer sentences). In our experiment, aggressive CAs had shorter sentences compared to cooperative ones but participants wrote lengthier inputs and engaged in longer conversations with aggressive CAs. The difference between our results and those of Ruane et al. (2021) can be attributed to the different contexts of the experiments. Here, the conversations took place within the context of a detective computer game (i.e., a narrative experience), whereas in Ruane et al. (2021) experiment, participants answered chatbots’ questions regarding the experience they have about university life. It might be that aggressive CAs trigger suspicion in users’ minds, raising their motivation to understand CAs’ attitude, resulting in longer sentences, and engaging in lengthier conversations to achieve their goals. Furthermore, the aggressive communication style emerged as the only predictor of participants’ identification of the culprit, highlighting the stronger influence of communication style over initial expectations. Aggressive CAs were significantly more frequently identified as the culprit, indicating an implicit association between aggressiveness and guiltiness (Infante and Wigley, 1986).

Finding 4 showed the influence of CAs’ communication style on participants’ conversation preferences. Participants preferred conversing with cooperative CAs resulting in longer conversations, regardless of their role in the narrative. These results align with the hypothesis made by Ruane et al. (2021) regarding participants’ engagement and the length of the conversations with CAs. Furthermore, participants’ ratings outlined the importance of CAs’ predictability on their conversations’ ranking. In narrative experiences, Loyall (1997) highlighted the importance of predictability for participants to anticipate their interaction based on their expectations as it enhances their enjoyment. In the current experiment, preferred conversations were those perceived as highly predictable and cooperative. To enhance participants’ preferences, CAs’ designers in narrative experience should manipulate CAs’ roles and communication styles to reduce the gap between users’ expectations and the conversation’s tone.

Finding 5 highlights the effect of the coherence between CAs’ role and their communication style on participants’ experience. In the experiment, the personality of cooperative suspects was rated significantly lower compared to the other conditions. Participants were inclined to accuse and argue with suspects, but the cooperative suspects surpassed their expectations by defusing tensions and responding calmly to their accusatory inquiries. This finding is consistent with Magerko (2007) perspective on defining believability as a metric for artificial agents, wherein participants evaluate them based on their expectations. Loyall (1997) further explains that the dimension of personality in believability attribution is not solely an assessment of the agent’s behavior but also reflects users’ acknowledgment of the agent’s distinctiveness, serving as a strong motivator for their engagement. As a result, suspects with an unexpected communication style receive lower rankings in terms of personality attribution, which could potentially hinder their effectiveness as engaging characters in a narrative experience.

The results of this experiment outline the importance of the role and communication style of CAs on both participants’ evaluation of CAs and their behaviors during the conversations. Participants perceived suspects as more intelligent than witnesses, but this result can be attributed to the different conversational strategies they used during their interactions. Furthermore, suspects with unexpected communication styles, such as being friendly, received lower ratings in terms of personality, potentially leading to a decrease in user engagement in the narrative experience. Aggressive communication style was highlighted as a significant predictor of being named as the culprit, regardless of the role. However, it’s important to consider the context of the crime-solving game as a potential limit for our findings as it could have influenced participants’ experiences during their conversations. Participants endorsing the role of a detective were actively seeking to identify a culprit, raising their motivation to interact with aggressive CAs. Hence, this engagement may differ in other social contexts. For example, future work should explore the effects of roles and communication styles in more diverse contexts, such as casual interactions in games (e.g., interaction with a random encounter), or in specific scenarios like interactions with sellers in role-playing games. Investigating these different contexts will undoubtedly deepen our understanding of the relationship between users’ expectations and CAs’ communication styles in narrative experiences. Additionally, as all CAs in our experiment were male, participants’ gender could have influenced their expectations and conversations with them. In future research, exploring the impact of participants’ gender on their perception and behaviors during conversations could provide a more comprehensive understanding of the relationships between their characteristics and CAs’ design parameters.

## 8 Conclusion

This experiment highlights the importance of considering users’ expectations in narrative experiences. The approach adopted here allows manipulating the roles and communication styles of different CAs and gain insight on their impact on users’ behaviors and on their perception of intelligence and believability. The information gathered from the experiment is crucial for creating engaging CAs that effectively convey the narrative through their interactions with users. By understanding the impact of factors such as roles, communication styles, and user expectations, developers can design CAs that enhance the immersive and interactive nature of the narrative, leading to a more enjoyable and compelling user experience. Precisely, manipulating the parameters of role and communication style affected participants’ experience during the conversations. The linguistic cues influenced participants’ behaviors and their perception of aggressiveness. These significant changes follow the studies made on the impact of personality traits on users’ experience with CAs. The results highlight the importance of communication style, regardless of the role, in identifying the culprit in a detective game. However, the role itself plays a crucial role in shaping users’ expectations and their attitude toward the CA. These findings have broader implications for the design of CAs in different narrative contexts, outlining the importance of attentively considering users’ expectations and perceptions. By carefully aligning roles and communication styles, developers can create more immersive and engaging experiences for users in various narrative scenarios. These results can be extended to other types of agents, such as embodied conversational agents. All types of agents, whether they are virtual assistants, chatbots, or even virtual characters in games, can greatly benefit from considering users’ expectations regarding their non-verbal behaviors. By incorporating appropriate non-verbal cues, such as gestures, facial expressions, and body language, agents can enhance the user experience and increase engagement. When combined with suitable roles and communication styles, this holistic approach can create more believable and immersive interactions, leading to improved user satisfaction and enjoyment.

## Data availability statement

The raw data supporting the conclusions of this article will be made available by the authors, without undue reservation.

## Ethics statement

The research conducted adhered strictly to the General Data Protection Regulation (GDPR) guidelines, validated and implemented by Ubisoft’s legal department. Only age, gender, and gaming habits were collected from participants for the sole purpose of research. The data collection process ensured compliance with GDPR regulations, emphasizing the confidentiality and privacy of individuals’ information. All procedures were conducted in accordance with the Declaration of Helsinki and aligned with local legislation and institutional requirements. Participants provided explicit written consent prior to their involvement in the study, highlighting the voluntary nature of participation and their ability to withdraw at any given time.

## Author contributions

RP: Writing – original draft, Writing – review and editing. MM: Writing – review and editing. CP: Writing – review and editing. MA: Writing – review and editing, Writing – original draft.
